# The Prevalence of Nonalcoholic Fatty Liver Disease and Relationship with Serum Uric Acid Level in Uyghur Population

**DOI:** 10.1155/2014/393628

**Published:** 2014-01-02

**Authors:** Wen Cai, Jiang-mei Song, Bei Zhang, Yu-ping Sun, Hua Yao, Yue-xin Zhang

**Affiliations:** ^1^School of Nursing, Xinjiang Medical University, No. 168 Youhao South Road, Urumqi, Xinjiang 830000, China; ^2^Department of Infectious Diseases, The First Affiliated Hospital of Xinjiang Medical University, Urumqi, Xinjiang 830054, China; ^3^School of Basic Medical Sciences of Xinjiang Medical University, No. 137 Liyushan South Road, Urumqi, Xinjiang 830054, China; ^4^Clinical Medical Research Institute, The First Affiliated Hospital of Xinjiang Medical University, No. 137 Liyushan South Road, Urumqi, Xinjiang 830054, China

## Abstract

*Objective*. To investigate the prevalence of nonalcoholic fatty liver disease (NAFLD) and the association of serum uric acid level with NAFLD in Uygur people, Xinjiang. *Methods*. A total of 2241 Uyghur persons (1214 males and 1027 females) were interviewed for physical checkups from 2011 to 2012. The clinical data of questionnaire survey, body mass index (BMI), abdominal circumference, blood pressure, blood sugar, blood lipid, and serum uric acid level were collected for analysis. *Results*. The prevalence rates of NAFLD determined by abdominal ultrasound examination and hyperuricemia were 43.9% and 8.4%, respectively. The persons with NAFLD had significantly higher serum uric acid levels than those without NAFLD (320 ± 88 versus 254 ± 80 **μ**mol/L; *P* < 0.001). The prevalence rate of NAFLD was significantly higher in subjects with hyperuricemia than that in those without hyperuricemia (78.19% versus 40.83%; *P* < 0.001), and the prevalence rate increased with progressively higher serum uric acid levels (*P* < 0.001). Multiple regression analysis showed that hyperuricemia was associated with an increased risk of NAFLD (odds ratio (OR): 2.628, 95% confidence interval (CI): 1.608–4.294, and *P* < 0.001). *Conclusion.* Serum uric acid level was significantly associated with NAFLD, and the prevalence rate of NAFLD increased with progressively higher serum uric acid levels.

## 1. Introduction

Nonalcoholic fatty liver disease (NAFLD) is formed by the accumulation of fat vacuoles in the cytoplasm of liver cells, ranging from simple steatosis to nonalcoholic steatohepatitis (NASH) and cirrhosis [1]. As the change of lifestyle and diet structure, increased incidence of NAFLD had become a serious public health problem in the world. It is not only a major killer in western countries, but also the serious health problem in China. NAFLD not only could directly lead to liver failure and hepatocellular carcinoma, but also participate in type 2 diabetes and atherosclerotic cardiovascular diseases [2]. NAFLD is complex and challenging for the human health.

Uric acid was the major end product of purine metabolism [3]. Mounting evidence suggests that elevated serum uric acid (SUA) frequently associates with the development or progression of metabolic syndrome (MS) [4, 5]. Nonalcoholic fatty liver disease was considered a hepatic manifestation of the metabolic syndrome. SUA was the body's main antioxidant [6]. Oxidative stress and lipid peroxidation injury were one of the important pathogenesis of NAFLD [7]. However, the relationship between SUA and NAFLD is still controversial, therefore, the research on the relationship between SUA and NAFLD has important significance for the prevention and diagnosis of NAFLD.

Studies had shown that uric acid might play an important role in the NAFLD [8–13]. A few cross-sectional studies in Korean adults and in nondiabetic adults from the USA had shown that elevated uric acid level was independently associated with ultrasound-diagnosed NAFLD [14, 15]. But recent study had shown that there was no correlation; Brazilian study had shown high levels of uric acid were not associated with NAFLD in overweight or obese children and adolescents [16]. The conflicting study might be the result of different sample sizes, different races in study populations, and differences in lifestyle and eating habits.

The research for employees and nondiabetic men in China had demonstrated a significant correlation between SUA level and NAFLD [17, 18], but the study population of the studies was Chinese Han population. Xinjiang region is one of the main provinces in China. Uyghur, as one of the main ethnic minority in Xinjiang, has unique eating habits and lifestyles [19]. Studies had shown that the prevalence of NAFLD was higher in the Uyghur when compared with the local Han population [20], but their SUA level and the prevalence rates of hyperuricemia were lower [21]. This study aims to explore the correlation between SUA and NAFLD in the Uyghur.

## 2. Materials and Methods

### 2.1. Research Object

A cross-sectional study was conducted among Uyghur people in physical examination group of Urumqi (China) to evaluate the relationship between the SUA level and NAFLD. Subjects who voluntarily visited the Health Promotion Center, the first affiliated hospital of Xinjiang Medical University, for a routine health checkup from January 2011 to December 2012. A total of 3378 Uyghur people were surveyed. 1137 subjects were excluded due to the following reasons: 326 subjects with positive serologic markers for hepatitis B or C virus; 489 subjects with heavy drinking (daily alcohol intake of 140 g/week or more); 59 subjects with liver cirrhosis; 34 subjects with abnormal dilatation of the biliary tree; 19 subjects with a history of malignancy; 69 subjects with a history of cardiovascular disease; 23 subjects currently using lipid-lowering drugs; 118 subjects with missing baseline data on medical information and drinking alcohol. There were 690 males and 447 females. The age was from 20 years to 70 years; the average age was 43.6 ± 8.8 years in the excluded crowd. The final sample size was 2241 participants; there were 1, 214 males and 1, 027 females. The age was from 20 years to 70 years; the average age was 43.2 ± 10.4 years. The comparisons on demographics between subjects and dropoffs population were shown in Table 1.

### 2.2. Method 

#### 2.2.1. Physical Examination

Questionnaire was compiled by combining with the risk factors of fatty liver according to the domestic literature. This research included age, sex, occupation, degree of education, income, past history of disease, genetic disease, smoking, drinking, exercise, sleep, diet, alcohol, and drugs. The research object was investigated with informed consent. Besides, the questionnaires were distributed uniformly by the investigators and filled out by the object of study on the spot, completed questionnaire independently. Their height, body mass, waist circumference, systolic blood pressure (SBP), and diastolic blood pressure (DBP) were measured according to the standard method for regular medical examination.

#### 2.2.2. Biochemical Determination

The serum from Uyghur persons collected in the morning was measured by the Hitachi 7060 Automatic Biochemical Analyzer to detected for SUA, triglyceride (TG), total cholesterol (TC), high-density lipoprotein cholesterol (HDL-C), low density lipoprotein cholesterol (LDL-C), fasting plasma glucose (FPG), alanine aminotransferase (ALT), aspartate aminotransferase (AST), urea nitrogen (BUN) and serum creatinine (SCr).

#### 2.2.3. Liver Ultrasound

An ultrasonographic diagnosis of fatty liver was defined as the presence of a diffuse increase of fine echoes in the liver parenchyma compared with the kidney or spleen parenchyma. Ultrasonographic diagnosis of fatty liver was determined by the radiologists using live images. The Uyghur persons on an empty stomach were examined by ultrasonography (Japan Siemens CDUS512 color ultrasonic diagnostic instrument, convex array probe, the frequency of 2~5 mHz). Two senior imaging specialists used the same ultrasonic diagnostic instrument to perform and issue a report. Imaging doctors did not know the examinee history and the study during the ultrasonic examination. If the diagnosis was different by two imaging doctors, a third imaging doctor was invited to diagnose.

### 2.3. Diagnostic Criteria

The diagnosis of NAFLD refers to the diagnostic criteria of nonalcoholic fatty liver disease diagnosis and treatment guidelines (revised edition) made by the Chinese Medical Association Branch of Hepatology in 2010 [22]. NAFLD was diagnosed by abdominal ultrasound. Subjects meeting any of the following criteria were excluded: subjects with an alcohol intake of 140 g/week or more for men and 70 g/week or more for women; subjects with a positive test for hepatitis B antigens or hepatitis C antibodies; subjects with serum creatinine ≥ 123.7 mmol/L; subjects with history of cancer, respiratory, renal, hepatobiliary, gout, and other rheumatologic diseases. The diagnostic criteria of hyperuricemia were SUA > 420 *μ*mol/L in male and SUA > 360 *μ*mol/L in women [23]. All of them did not take any medication or antioxidants.

The diagnostic criteria of the metabolic syndrome (MS) must be in accordance with three of the following components or all [24]: (1) the central obesity: waist > 90 cm in men and >80 cm in women or body mass index (BMI) > 25.0 kg/m^2^; (2) high blood TG: fasting plasma TG ≥ 1.7 mmol/L; (3) the level of HDL-C: HDL-C < 1.03 mmol/L in man and HDL-C < 1.29 mmol/L in women; (4) blood pressure: SBP ≥ 130 mmHg (1 mmHg  =  0.133 kPa) or DBP ≥ 85 mmHg or had been diagnosed with high blood pressure; (5) plasma glucose: FPG ≥ 5.6 mmol/L or had been diagnosed with type 2 diabetes.

### 2.4. Statistical Methods

Data were analyzed using SPSS software for Windows version 13.0. Measurement data is expressed by ($\overset{-}{x}\pm s$); the mean between the two groups was compared by *t*-test, and the comparison of multiple mean with analysis of one-way ANOVA and Chi-square test was adopted to compare the percentage or count data. For the correlations between NAFLD and SUA, MS were analyzed by multivariable logistic regression analysis (forward wald, entry: 0.05, removal: 0.10), inspection level *α* = 0.05. This study was approved by the Ethics Committee of the First Affiliated Hospital of Xinjiang Medical University and was conducted according to the standards of the Declaration of Helsinki. Written informed consent was obtained from the participants (20120220-135).

## 3. Results

### 3.1. Clinical Features in Uyghur Persons

A total of 984 cases (43.90%) were diagnosed of NAFLD in 2, 241 Uyghur persons. The 188 persons (8.38%) had hyperuricemia. 769 persons (34.30%) had MS. 1475 cases (65.8%) had central obesity, 721 cases (65.8%) high TG levels, 841 cases (37.5%) had low HDL-C, 820 cases (36.6%) had increased blood pressure, and 407 cases (18.2%) had fasting blood glucose. The age, blood pressure, body mass index, ALT, AST, SCr, FBG, TC, TG, LDL-C, and SUA level in persons with NAFLD were higher than those in persons without NAFLD, but the level of HDL-C, drinking, and exercise were relatively low in NAFLD persons; the difference had statistical significance (*P* < 0.05); the difference of drinking and exercise had no statistical significance between NAFLD persons and persons without NAFLD (see Table 2).

### 3.2. The Correlation between SUA Level and NAFLD

**Figure 1:**
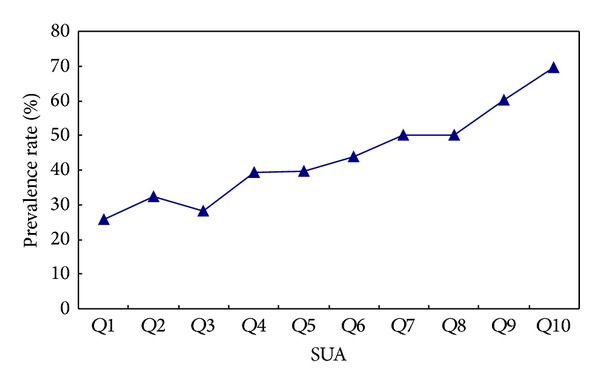
Prevalence rate of NAFLD in the subjects with different deciles levels of serum uric acid.

78.19% of 147 persons with hyperuricemia and 40.83% of 832 persons without hyperuricemia were diagnosed for NAFLD; the difference was statistically significant (*χ* ^2^ = 98.020, *P* < 0.05). Spearman correlation analysis showed that there was positive correlation between NAFLD and SUA level (*r* = 0.365, *P* < 0.001). In order to further explore the relationship between SUA and NAFLD, the SUA level was grouped by deciles (shown in Table 3). The OR values of Q2, Q3, Q4, Q5, Q6, Q7, Q8, Q9, and Q10 groups were 1.374, 1.127, 1.873, 1.901, 2.267, 2.837, 2.836, 4.309, and 6.524, when compared with group Q1 (see Table 4). The prevalence rate of NAFLD as shown in Figure 1, the tended to increase with the increase of SUA level.

### 3.3. NAFLD Risk Factor Analysis

Multivariate logistic analysis was performed to evaluate risk factors for NAFLD. The results showed that BMI, SUA, HDL-C, TG, TC, FPG, and BP had effects on NAFLD, and it was statistically significant (shown in Tables 5 and 6).

## 4. Discussion

NAFLD is a common chronic liver disease with genetic, environmental, metabolic, and stress-related components. It now affects 20–30% of the general population in North America and similar countries. The median prevalence of ultrasonographic steatosis in Chinese populations was 10% but ranged from 1% to more than 30% [25]. The prevalence of NAFLD in Uyghur persons was 43.9% in this study; it was higher than that of Han persons in Chengdu, Southwest China [26], and it was consistent with other studies [27]. This may be related to the following reasons: the Uyghur have different genetic background, lifestyle, and eating habits. They are fond of wheat, meat, cheese, condensed milk, butter, and high fat diet but seldom eat fresh fruits and vegetables. Meanwhile, overweight and central obesity in the Uyghur people are common; the detection rate of centrality obesity in Uyghur people was as high as 65.8%, and similar studies had also shown that prevalence of centrality obesity in Uygur people was higher than Han people in Xinjiang [28]. Moreover, most of the objects of this study were civil servants or office workers who were mental workers and lacked enough physical exercise.

The SUA level and hyperuricemia prevalence in Uyghur people were lower than the Han people [29]. BMI of Uyghur was significantly higher than that of the Chinese Han, but the SUA level of Uyghur was lower than that of the Chinese Han in this study. The result was inconsistent with the previous results in which SUA was increased with the increase of BMI. The reason may be related to the unique genetic background in Uyghur, and the genetic studies are needed in Uyghur.

This research showed that the level of SUA in Uyghur population was low, but the risk of NAFLD in Q10 was 6.524 times, as compared to the subjects with Q1, which suggested that SUA level was associated with NAFLD and may be one of the pathogenesis of NAFLD. With the increase of the level of uric acid and hemoglobin, the prevalence rate of NAFLD was increased. The results of previous studies are consistent with those of our study. Li et al. reported that serum uric acid level was associated with NAFLD in 8925 employees of chemical company [17]. In addition, a cohort study reported that serum uric acid was an independent predictor for developing ultrasonographically detected fatty liver even in normal-weight men [2]. Our results suggested that those nine factors are closely associated with the risk for NAFLD. Notably, hyperuricemia was found to be an important risk factor for NAFLD.

The pathogenesis of NAFLD remains unclear, but current understanding of the progression of NAFLD involves the “2-hit hypothesis.” The association between SUA and NAFLD might be explained by the theory. The “first hit” is excessive fat accumulation in hepatocytes, which is closely linked to insulin resistance and obesity. In this process, insulin resistance promotes lipolysis of peripheral adipose tissue and increases free fatty acid influx into the liver, and it leads to hyperinsulinemia, which increases uric acid synthesis and reduces the renal excretion of uric acid [30]. The ‘‘second hit” is the process from oxidative stress to hepatocyte injury, inflammation, and fibrosis; SUA has been proved to be proinflammatory and an increased SUA level reflects the rate of cell turnover, which itself may be a part of the inflammatory process [31]. However, uric acid had long been recognized as a natural cleaner of oxidative stress product; a recent study showed that treatment with uric acid in obese ob/ob mice resulted in a nearly complete resolution of fatty liver [32]. It was speculated that SUA level might be the physiological compensatory mechanism for enhancing patients with NAFL against oxidative stress. In recent years, some study found that SUA had strong antioxidant function in the body of patients with MS [33]. Although the regulating mechanism of SUA in the redox reaction balance was not yet clear, but in NAFLD patients SUA may play a contradiction opposite role in oxidative stress [34]. Serum uric acid level was significantly associated with NAFLD, but future studies need to explain the physiological mechanism for this association.

The deficiency in this study is that most of the research objects in this study were civil servants or office workers; their prevalence of hyperuricemia and NAFLD may be higher than that in community or rural area. Meanwhile, the study does not define causal relationships between serum uric acid and NAFLD, a challenge that will require further study.

## 5. Conclusion

In a word, increased SUA level is closely associated with NAFLD in Uyghur people. The SUA level in clinical testing was an effective auxiliary examination for assessing the risk of NAFLD.

## Figures and Tables

**Table 1:** The comparisons on demographics between subjects and drop-offs.

	Subjects	Drop-offs	*t*/*χ* ^2^	*P*
Gender (male/female)	1214/1027	690/447	4.204	0.040
Age	43.2 ± 10.4	43.6 ± 8.8	1.057	0.291
(Body mass index) BMI	26.27 ± 4.29	26.98 ± 7.38	−2.744	0.006

**Table 2:** Comparison of clinical and laboratory indexes between NAFLD and non-NAFLD status.

Clinical indicator	Non-NAFLD group (*n* = 1257)	NAFLD group (*n* = 934)	*t*(*χ* ^2^) value	*P* value
Age (year)	40.9 ± 10.2	46.0 ± 10.0	−11.829	<0.05
Gender (male/female)	538/719	676/308	149.123	<0.05
BMI (kg/m^2^)	24.7 ± 3.3	29.8 ± 9.7	−16.744	<0.05
SBP (mm Hg)	119 ± 34	130 ± 17	−8.925	<0.05
DBP (mm Hg)	74 ± 12	82 ± 12	−14.798	<0.05
FPG (mmol/L)	5.0 ± 1.0	5.6 ± 1.6	−11.715	<0.05
TG (mmol/L)	1.3 ± 1.1	2.2 ± 1.6	−15.864	<0.05
TC (mmol/L)	4.7 ± 1.9	5.2 ± 1.8	−6.347	<0.05
HDL-C (mmol/L)	1.33 ± 0.35	1.14 ± 0.39	11.141	<0.05
LDL-C (mmol/L)	2.79 ± 0.77	3.16 ± 0.74	−10.859	<0.05
BUN (mmol/L)	4.8 ± 1.5	5.0 ± 1.4	−3.879	<0.05
Scr (mmol/L)	66 ± 20	71 ± 16	−6.083	<0.05
SUA (*μ*mol/L)	254 ± 80	320 ± 88	−18.407	<0.05
AST (mmol/L)	20 ± 10	24 ± 18	−7.259	<0.05
ALT (mmol/L)	23 ± 10	35 ± 26	−13.7	<0.05
Smoking (never/yes/ever)	804/339/114	523/359/102	27.900	<0.05
Drinking (never/occasional)	686/571	522/462	0.517	0.472
Exercise (sedentary/moderate/active)	610/325/322	478/274/232	2.093	0.351

*Abbreviations*: BMI: body mass index, SBP: systolic blood pressure, DBP: diastolic blood pressure, FPG: fasting blood glucose, TG: triglyceride, TC: total cholesterol, HDL-C: high density lipoprotein cholesterol, LDL-C: low density lipoprotein cholesterol, SCr: serum creatinine, AST: glutamate aminotransaminase, and ALT: alanine transaminase.

**Table 3:** The SUA level grouped by deciles.

SUA level	Males	Females
*Q*1	≤235.35 *μ*mol/L	≤156.00 *μ*mol/L
*Q*2	235.36~262.70 *μ*mol	156.01~174.16 *μ*mol
*Q*3	262.71~285.10 *μ*mol	174.17~190.56 *μ*mol
*Q*4	285.11~306.00 *μ*mol	190.57~205.62 *μ*mol
*Q*5	306.01~329.00 *μ*mol	205.63~219.64 *μ*mol
*Q*6	329.01~346.05 *μ*mol	219.65~234.24 *μ*mol
*Q*7	346.06~369.00 *μ*mol	234.25~250.00 *μ*mol
*Q*8	369.01~396.27 *μ*mol	250.01~271.78 *μ*mol
*Q*9	396.28~432.23 *μ*mol	271.79~308.80 *μ*mol
*Q*10	>432.23 *μ*mol	>308.80 *μ*mol

**Table 4:** Association of SUA level with NAFLD.

SUA level	Case	NAFLD (*n* (%))	*χ* ^2^ value	*P*	OR (95% CI of OR)
*Q*1	225	58 (25.78)	—	—	1
*Q*2	223	72 (32.29)	2.301	0.129	1.374 (0.911, 2.071)
*Q*3	224	63 (28.13)	0.315	0.575	1.127 (0.742, 1.711)
*Q*4	226	89 (39.38)	9.409	0.002	1.873 (1.254, 2.797)
*Q*5	224	89 (39.73)	9.832	0.002	1.901 (1.272, 2.840)
*Q*6	225	99 (44.00)	16.189	<0.001	2.267 (1.522, 3.377)
*Q*7	228	114 (50.00)	26.593	<0.001	2.837 (1.909, 4.217)
*Q*8	218	109 (50.00)	26.054	<0.001	2.836 (1.900, 4.232)
*Q*9	224	135 (60.27)	50.848	<0.001	4.309 (2.884, 6.438)
*Q*10	224	156 (69.64)	79.179	<0.001	6.524 (4.316, 9.861)

**Table 5:** The values of the influence factor of NAFLD.

Variable	Assignment
Sex	0 = female; 1 = male
Age	0 = 20–40 years, 1 = 41–59 years, 2 = 60–70 years
BMI	0 = BMI < 25 kg/m^2^; 1 = BMI ≥25 kg/m^2^
BP	0 = normal; 1 = hypertension
SUA	0 = normal; 1 = hyperuricemia
TG	0 = <1.7 mmol/L; 1 = ≥1.7 mmol/L
TC	0 = <5.72 mmol/L; 1 = ≥5.72 mmol/L
HDL-C	0 = normal; 1 = <0.9 mmol/L (male), <1.0 mmol/L (female)
LDL-C	0 = normal; 1 = ≥5.72 mmol/L
AST	0 = ≤40 U/L; 1 = >40 U/L
ALT	0 = ≤40 U/L; 1 = >40 U/L
FPG	0 = <6.10 mmol/L; 1 = ≥6.10 mmol/L
Smoking	0 = no smoking; 1 = yes; 2 = quit smoking
Drinking	0 = no drinking; 1 = occasional
Exercise	1 = <1 h/week (sedentary); 2 = 2-3 h/week (moderate) 3 = ≥3 h/week (active)

*Abbreviations*: BMI: body mass index, BP: blood pressure, SUA: serum uric acid, TG: triglyceride, TC: total cholesterol, HDL-C: high density lipoprotein cholesterol, LDL-C: low density lipoprotein cholesterol, AST: glutamate aminotransaminase, ALT: alanine transaminase, and FPG: fasting blood glucose.

**Table 6:** The risk factors of NAFLD in Multivariate logistic regression analysis.

	*β*	SE	Wald	df	*P*	OR	95% CI for OR	
Age	0.179	0.071	6.319	1.000	0.012	1.196	1.040	1.374
BMI	2.130	0.162	173.041	1.000	0.000	8.412	6.125	11.553
BP	0.487	0.129	14.222	1.000	0.000	1.628	1.264	2.097
FPG	0.581	0.162	12.823	1.000	0.000	1.788	1.301	2.457
TG	0.750	0.141	28.511	1.000	0.000	2.118	1.608	2.789
TC	0.565	0.136	17.284	1.000	0.000	1.760	1.348	2.297
HDL-C	0.599	0.128	21.810	1.000	0.000	1.821	1.416	2.342
AST	0.916	0.312	8.598	1.000	0.003	2.500	1.355	4.612
SUA	0.966	0.251	14.876	1.000	0.000	2.628	1.608	4.294
Constant	−3.168	0.206	237.568	1.000	0.000	0.042		

*Abbreviations*: BMI: body mass index, BP: blood pressure, SUA: serum uric acid, TG: triglyceride, TC: total cholesterol, HDL-C: high density lipoprotein cholesterol, LDL-C: low density lipoprotein cholesterol, AST: glutamate amino transaminase, ALT: alanine transaminase, and FPG: fasting blood glucose.
